# Gene expression reprogramming of Pseudomonas alloputida in response to arginine through the transcriptional regulator ArgR

**DOI:** 10.1099/mic.0.001449

**Published:** 2024-03-21

**Authors:** María Antonia Molina-Henares, María Isabel Ramos-González, Serena Rinaldo, Manuel Espinosa-Urgel

**Affiliations:** 1 Department of Biotechnology and Environmental Protection, Estación Experimental del Zaidín, CSIC. Profesor Albareda, 1. Granada 18008, Spain; 2 Laboratory Affiliated to Istituto Pasteur Italia - Fondazione Cenci Bolognetti - Department of Biochemical Sciences “A. Rossi Fanelli”, Sapienza University of Rome, Rome, Italy

**Keywords:** gene expression, regulation, arginine, metabolism, redox

## Abstract

Different bacteria change their life styles in response to specific amino acids. In *Pseudomonas putida* (now *alloputida*) KT2440, arginine acts both as an environmental and a metabolic indicator that modulates the turnover of the intracellular second messenger c-di-GMP, and expression of biofilm-related genes. The transcriptional regulator ArgR, belonging to the AraC/XylS family, is key for the physiological reprogramming in response to arginine, as it controls transport and metabolism of the amino acid. To further expand our knowledge on the roles of ArgR, a global transcriptomic analysis of KT2440 and a null *argR* mutant growing in the presence of arginine was carried out. Results indicate that this transcriptional regulator influences a variety of cellular functions beyond arginine metabolism and transport, thus widening its regulatory role. ArgR acts as positive or negative modulator of the expression of several metabolic routes and transport systems, respiratory chain and stress response elements, as well as biofilm-related functions. The partial overlap between the ArgR regulon and those corresponding to the global regulators RoxR and ANR is also discussed.

## Data Summary

The authors confirm all supporting data, code and protocols have been provided within the article or through supplementary data files.

## Introduction

Many bacteria have the capacity to build multicellular communities associated to solid surfaces and protected by a self-produced extracellular matrix, known as biofilms [1,3]. The transition between planktonic and sessile existence involves changes in gene expression and metabolism of bacterial cells, associated with variations in the intracellular levels of the second messenger cyclic diguanylate (c-di-GMP) [4,5]. These, in turn, are modulated in response to chemical environmental and metabolic cues, as well as physical stimuli [6,8]. Among the molecules that influence c-di-GMP signalling and biofilm development, a noteworthy role has been ascribed to certain amino acids. In particular, arginine has emerged as an important chemical indicator that regulates multicellular behaviours in different bacteria, such as *Pseudomonas aeruginosa*, *Burkholderia cenocepacia* or *Salmonella typhimurium*, through the activity of proteins involved in c-di-GMP turnover [9,12].

Arginine has also been shown to play a key role in *Pseudomonas putida* KT2440 (recently reclassified as *Pseudomonas alloputida* KT2440 [13,14], although this name change has still not been commonly adopted in the literature nor in most databases), a biofilm-forming, plant-beneficial strain that has also been widely used in biotechnological engineering [15,17]. In this bacterium, arginine biosynthesis and transport modulate c-di-GMP levels and the associated phenotypic changes [18,19]. This response requires the transcriptional regulator ArgR [20], an AraC/XylS family regulator, which in different bacteria controls arginine metabolism and uptake in the presence of the amino acid [21,22]. In KT2440, *argR* is the last gene of an operon that encodes the main arginine transporter (PP_4486-PP_4483, annotated as *argT-hisPQM*) and the regulator (PP_4482) [19,20]. This transcriptional unit is expressed from a promoter upstream *argT* that responds to arginine and is dependent on ArgR, while a second arginine-inducible promoter with lower activity is present upstream *argR* and also contributes to its transcription (Fig. S1, available in the online version of this article; [20]). Therefore, ArgR positively controls arginine uptake and its own expression in response to the amino acid. Arginine biosynthesis, on the other hand, is negatively regulated by ArgR at least through *argG* [20], the gene encoding argininosuccinate synthase, which catalyses the second-to-last stage in the pathway.

Besides protein synthesis, its central metabolic role, and its impact on c-di-GMP, arginine has additional effects on *P. alloputida*, being important for the maturation of the siderophore pyoverdine and for oxidative stress tolerance, likely through the synthesis of polyamines [23], for which arginine is the precursor. Mutants presenting arginine auxotrophy are impaired in the release of pyoverdine and also show increased production of reactive oxygen species [23]. Furthermore, arginine influences the oxygen consumption and respiratory activity of KT2440, and ArgR is required to maintain the metabolic activity of cells during growth and biofilm development in the presence of the amino acid [24].

All these findings have led us to hypothesize that the intracellular contents of the amino acid act as an internal gauge of the overall metabolic and redox state of *P. alloputida* cells [20,25], which in turn could be decisive in the change or maintenance of lifestyles (planktonic versus biofilm). In this model, ArgR would have a central role, being responsible for the modulation of intracellular arginine pools. The evidence of a regulatory feedback loop between second messenger signalling and expression of ArgR, through the c-di-GMP-associated regulator FleQ, strongly supports this notion [20]. Additional elements, such as the phosphodiesterase RmcA, would contribute to fine-tuning c-di-GMP levels in response to arginine, in this case through direct interaction of the amino acid with a periplasmic domain of the protein [26].

While arginine metabolism and the role of ArgR have been studied in species such as *P. aeruginosa* and *Escherichia coli*, there is limited information on the molecular mechanisms by which *P. alloputida* adjusts its metabolism in response to arginine. The pleiotropic effects associated to the amino acid suggest that in this bacterium, ArgR could have a broad relevance, beyond its mere role as regulator of the transport and synthesis of arginine. In this work we present a global transcriptomic analysis that exposes a wide impact of ArgR on gene expression during growth in the presence of arginine. A partial overlap between the ArgR regulon and those of the global regulators ANR and RoxR is also unveiled.

## Methods

### Bacterial strains and growth conditions

*Pseudomonas putida* (*alloputida*) KT2440 and its Δ*argR* derivative have been described elsewhere [20,27]. Strains were routinely grown at 30 °C under orbital shaking in rich LB medium or M9 minimal medium [28] with 20 mM glucose as carbon source. Where indicated, 5 mM l-arginine (Merck) was added.

### RNA extraction

Cultures of KT2440 and its Δ*argR* derivative were grown in duplicate in M9 with glucose and 5 mM l-arginine for 7 h, to an OD600 ≈ 1.6, when expression of *argR* has been previously shown to be maximal [20]. Alternatively, RNA was extracted from cultures in the same medium but grown overnight in flasks with orbital shaking (aerobic conditions) or in sealed 50 ml tubes without aeration (microaerobic conditions).

Cells were recovered by centrifugation and immediately frozen in liquid nitrogen. Total RNA was extracted (three samples per biological replicate of each strain) with TRI-Reagent (Ambion, Austin, TX) according to the manufacturer’s protocol, except that the reaction mixture was preheated at 65 °C. Samples were incubated for 10 min at 65 °C, followed by treatment with RNase-free DNase I (Turbo RNA free; Ambion) plus RNaseOut (Invitrogen), followed by enzyme inactivation. RNA concentration was determined with a NanoDrop One^C^ spectrophotometer (Thermo Fisher).

### Transcriptomic analysis by RNASeq

After quality verification (Qubit and Bioanalyzer), RNA samples (100 ng) were used to construct libraries with Illumina Stranded Total RNA Prep with Ribo‐Zero Plus. RNA-Seq was carried out on an Illumina NextSeq apparatus, with a Mid output (150 cycles, 2×72 bases). A total of 80.52 and 78.22 million paired-end reads were obtained for the wild-type and mutant samples, respectively. Quality control, library preparation and RNA-Seq were performed at the Genomics Service of the Instituto de Parasitología y Biomedicina López-Neyra (Granada, Spain).

Data analysis was carried out with software freely available in the Galaxy platform (https://usegalaxy.org/; [29]). Genome-mapped BAM files from each of the 24 reads files were obtained with STAR [30], using the genome of *P. putida* KT2440 as reference (Genbank AE015451.2). Next, featureCounts [31] was used to compute the abundance of counts per gene in each sample. Finally, edgeR [32,33] was used for differential expression analysis, with the following parameters: filtering by minimum count per million, to remove genes with too low expression levels; log_2_ fold change ≥0.5/≤ −0.5; *P*-value threshold ≤0.05, with Benjamini-Hochberg adjustment (false discovery rate <0.05); and normalization by trimmed mean of M values (TMM). Functional annotations for the analysed loci were obtained from the *Pseudomonas* Genome Database (www.pseudomonas.com; [34]).

Transcriptomic analysis datasets are publicly available at the Galaxy platform in the following link: https://usegalaxy.org/u/manuel68/h/argr-p-putida. Reads files, counts per gene and other data are also available from the authors upon request.

### Quantitative real-time PCR (qRT-PCR)

RNA from cultures of KT2440 and its Δ*argR* derivative was extracted and purified as detailed above, using three biological replicates. RNA quality was verified by agarose gel electrophoresis, the RNA concentration was determined using a NanoDrop One^C^ spectrophotometer (NanoDrop Technologies), and the absence of any residual DNA was checked by PCR. Reverse transcription reactions were carried out according to the manufacturer’s protocol, using 0.5 mg RNA, SuperScript II reverse transcriptase (Invitrogen) and random hexamer primers to generate the corresponding cDNA.

Quantitative RT-PCR assays were performed using iCycler Iq (Bio-Rad). Template cDNAs from the three experimental and reference samples were amplified in triplicate using the primers listed in Table S1, designed to render PCR products of 125–220 bp. Each reaction mixture contained 1 µl of a dilution of the target cDNA (1 : 10 to 1 : 10 000) and 11.5 µl of Sybr Green mix (Molecular Probes). Samples were initially denatured by heating at 95 °C for 10 min, followed by a 40-cycle amplification and quantification programme (95 °C for 15 s, 62 °C for 30 s, and 72 °C for 20 s) with a single fluorescence measurement per cycle. To confirm the amplification of a single PCR product, a melting curve was obtained by slow heating from 60–99.5 °C at a rate of 0.5 °C every 10 s for 80 cycles, with continuous fluorescence scanning.

Gene expression results of the target genes were normalized relative to those obtained for the 16S rRNA gene using BIO-RAD iQ5 software. Quantification was based on the 2^−ΔΔCT^ method [35].

### Bioinformatic analysis of putative ARG boxes

To identify the presence of potential ARG boxes in the upstream regions of identified transcriptional units, the Virtual Footprint tool at the PRODORIC database [36] was initially used, searching the whole genome of KT2440 with the *P. aeruginosa* ArgR matrix in the database (with the consensus TGTCGCN_6_GNAA). The search was restricted to intergenic regions and a maximum distance of 300 bp from the ATG. WebLogo [37] was used to create graphical representations of consensus sequences.

## Results

### Transcriptomic analysis reveals a global influence of ArgR in the response to arginine

As mentioned in the Introduction, besides modulating c-di-GMP levels and biofilm formation, arginine supplementation alters the respiratory activity and oxygen consumption of *P. alloputida* KT2440, and the transcriptional regulator ArgR is required to sustain the metabolic activity of biofilms in the presence of the amino acid [24]. To gain further insights into the cellular roles of ArgR, a global transcriptional analysis was carried out by RNA-Seq, comparing the wild-type strain and a previously constructed Δ*argR* mutant [20] growing in minimal medium with glucose as carbon source and supplemented with 5 mM arginine, as described in the Methods section, to ensure maximal expression of *argR* in the wild-type strain, as indicated in the Introduction.

**Fig. 1.**
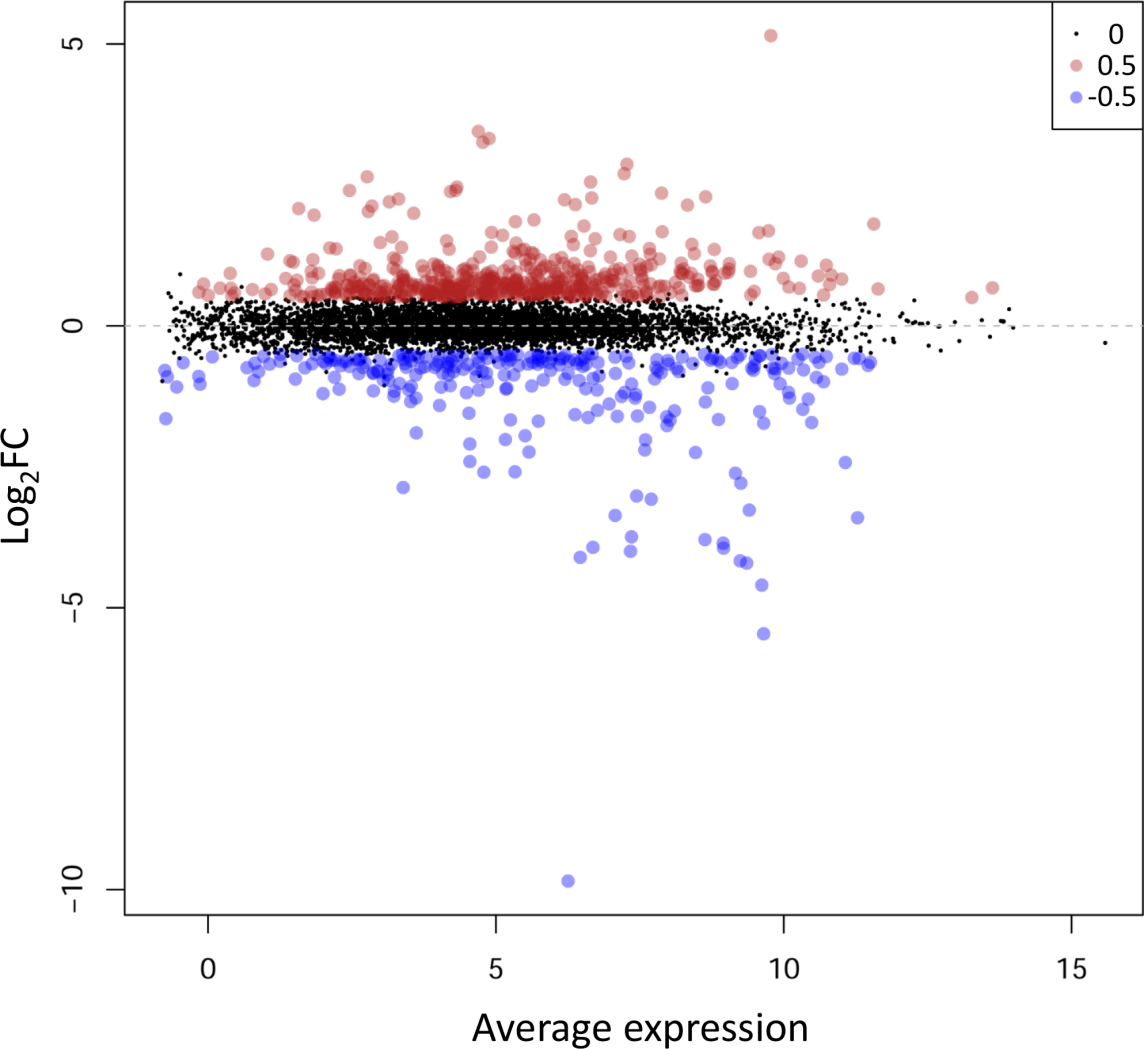
Mean difference (MD) plot of the RNA-Seq data corresponding to the Δ*argR* mutant vs. wild-type strain. Data above log_2_ fold change=0.5 or below log_2_ fold change=−0.5 (*P*-value≤0.05) are shown in red and blue, respectively.

With the default cutoff parameters used (log_2_ fold change ≥0.5/≤ −0.5; *P*-value threshold ≤0.05, with Benjamini-Hochberg adjustment, false discovery rate <0.05), a total of 497 up-regulated and 262 down-regulated genes were identified in the mutant with respect to the wild-type (Fig. 1). In general, no all-or-nothing effect was observed in terms of normalized number of reads per gene. More strict parameters (*P*-value<0.01; log2 fold change >0.8/ <−0.8) were used for increased confidence in the selection of functions influenced positively or negatively by ArgR. As detailed in Tables S2 and S3, 201 up-regulated and 118 down-regulated genes in the mutant (excluding *argR*, which is deleted and therefore appears as the most differentially expressed gene) with respect to the wild-type met these more stringent criteria. According to previous global analyses of the primary transcriptome of KT2440 growing in minimal medium with glucose or citrate as carbon source [38], these genes would correspond to 136 and 77 transcriptional units, respectively. In comparison, only 37 genes were identified as being regulated by ArgR in a transcriptomic analysis of *P. aeruginosa* using microarrays [39], whereas in *Streptomyces coelicolor*, expression of over 1500 genes is influenced by this transcriptional regulator [40].

**Fig. 2.**
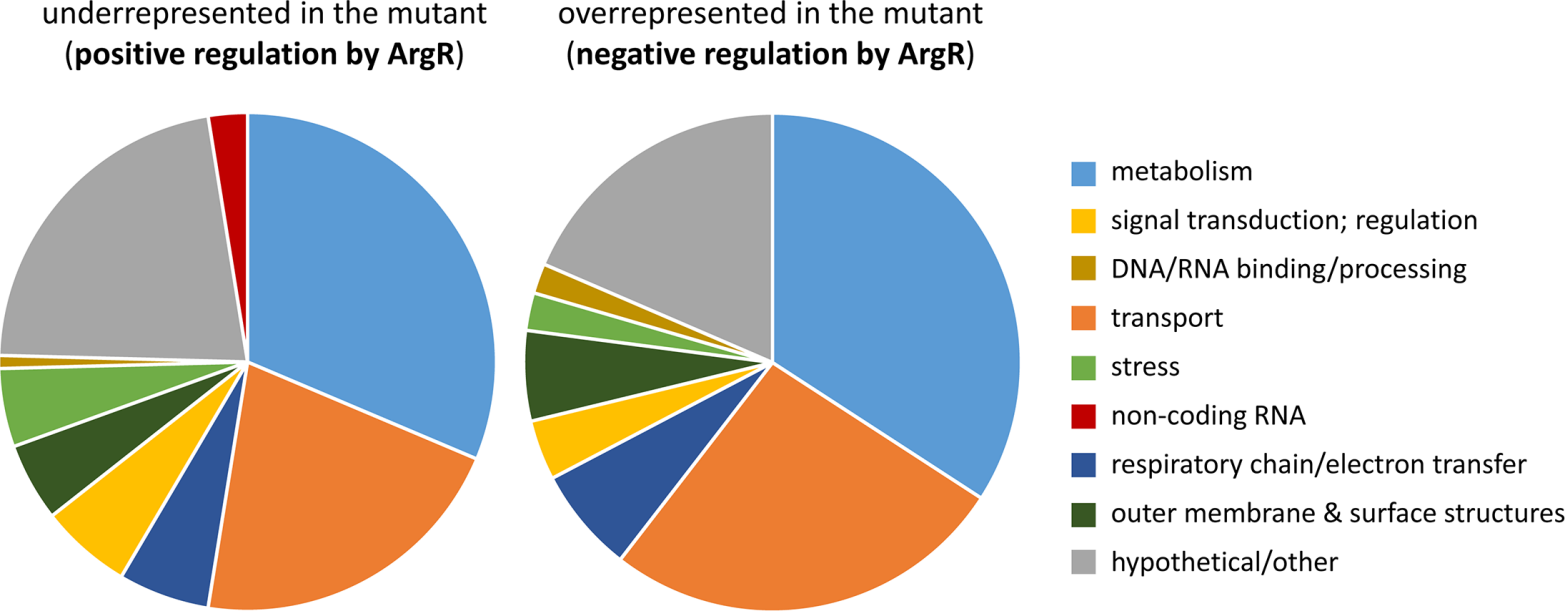
Functional classification and relative abundance of genes identified as positively or negatively regulated by ArgR in the RNA-Seq analysis (*P*-value<0.01; log_2_ fold change >0.8/ <−0.8). Details can be found in Tables S2 and S3.

**Fig. 3.**
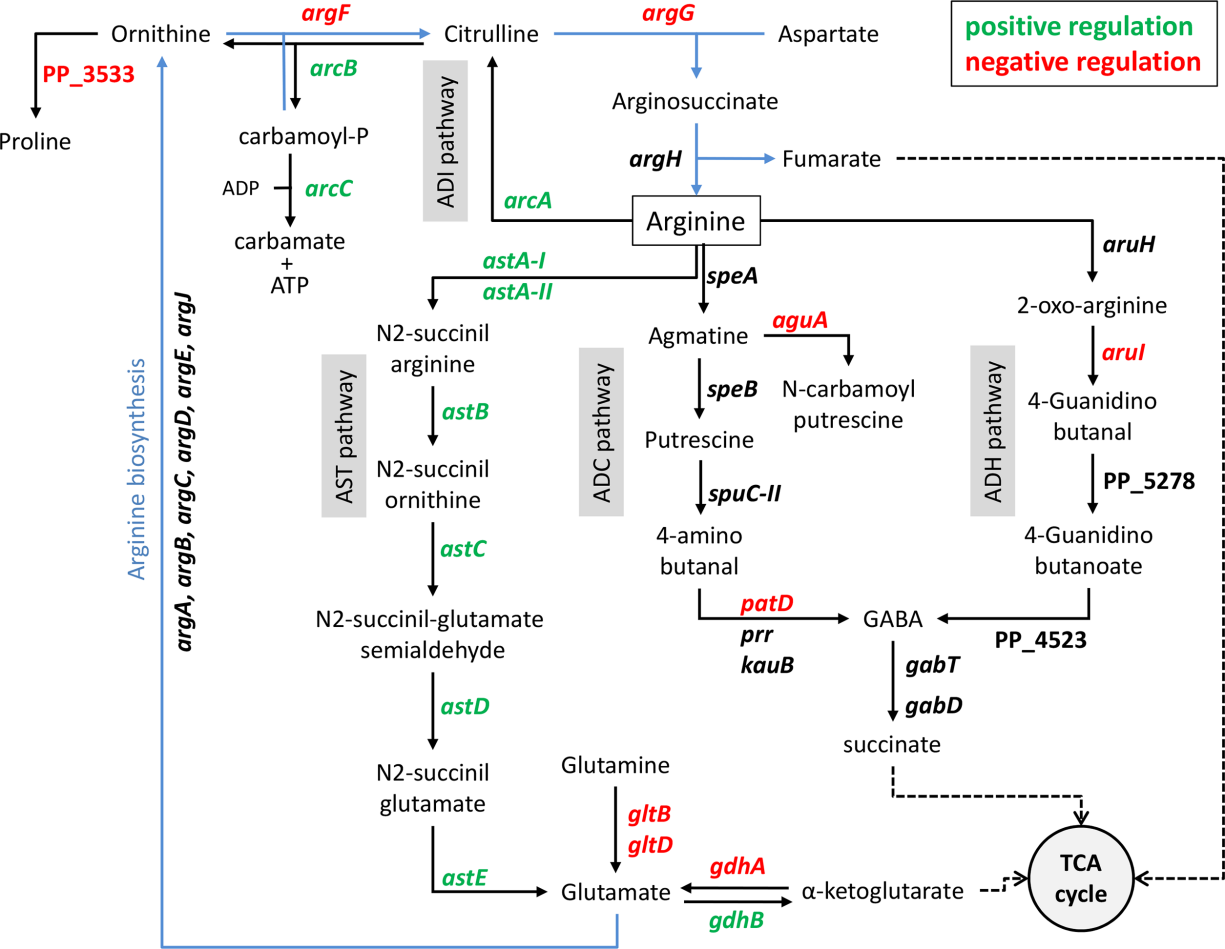
Influence of ArgR on arginine metabolism. Blue arrows correspond to the arginine biosynthesis pathway (some details have been omitted for simplicity), and black arrows to the different arginine catabolic pathways, indicated in the grey boxes, and other relevant reactions associated to arginine/glutamate metabolism. Broken arrows indicate compounds feeding into the TCA cycle. Elements positively or negatively regulated by ArgR are shown in green and red, respectively.

Fig. 2 summarizes the functional classification and relative abundance of the differentially expressed genes. As expected, genes involved in arginine transport or metabolism were among those showing differential expression. These include *argG* and *argT* (up- and down-regulated in the mutant, respectively), whose promoters have previously been shown to be oppositely regulated by ArgR [20]. Additional elements in the arginine synthesis pathway are negatively controlled by ArgR (Fig. 3), namely, *argF*, encoding anabolic ornithine carbamoyltransferase, and elements involved in the synthesis of glutamate, precursor of the biosynthesis of arginine. ArgR also modulates arginine catabolism in an intriguing way (Fig. 3). Genes corresponding to the arginine succinyltransferase (AST) and the arginine deiminase (ADI) pathways show increased expression in the wild-type, indicative of positive regulation by ArgR, whereas some of the steps in the arginine decarboxylase (ADC) and arginine dehydrogenase (ADH) pathways are overexpressed in the mutant, and therefore would be negatively regulated by ArgR. These results contrast with those obtained in *E. coli*, where ArgR regulates all the arginine biosynthesis and transport operons, but not the catabolic genes [22].

**Fig. 4.**
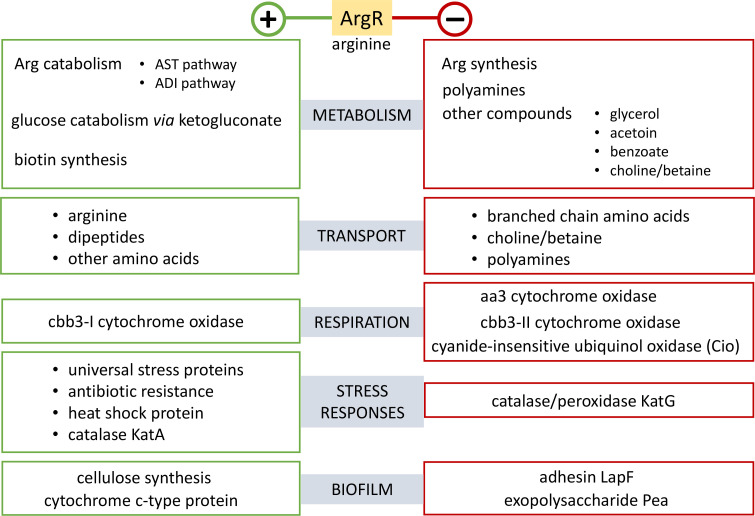
Summary of relevant functions belonging to the ArgR regulon.

A summary of the main elements influenced by ArgR is shown in Fig. 4. Besides arginine metabolism, genes related to other metabolic routes show differential expression in the Δ*argR* mutant with respect to the wild-type under the conditions tested. Benzoate and acetoin catabolism, for example, are negatively regulated by ArgR. On the other hand, positive regulation is exerted by ArgR on biotin biosynthesis genes and on enzymes involved in the conversion of gluconate to gluconate-6-P via ketogluconate, suggesting that ArgR modulates the ‘choice’ for glucose utilization pathways in *P. alloputida* during growth in the presence of glucose and arginine. It is also worth mentioning that along with the *argT*/*hisPQM* operon, the second most relevant arginine transporter, encoded by the predicted operon PP_0283(*aotP*)-PP_0282(*artJ*)-PP_0281-PP_0280 [19], is positively regulated by ArgR. Other amino acid and dipeptide transporters also show reduced mRNA levels in the Δ*argR* mutant. Additionally, a set of genes encoding proteins of the universal stress (USP) family, and two non-coding small RNAs would be positively modulated by ArgR.

Four out of the five terminal oxidases present in *P. alloputida* were found in our study to be differentially expressed in each genetic background. Two gene clusters encoding the elements of cbb3 cytochrome c oxidases (*ccoNOQP*-I and II) are positively and negatively regulated by ArgR, respectively (note that the number assigned to oxidases cbb3-1 and cbb3-2 is reversed in KT2440 with respect to *P. aeruginosa*). Furthermore, the cluster PP_0103-PP_0104-PP_0105-PP_0106, encoding the cytochrome c oxidase Aa3, and *cioB*, encoding the subunit II of cyanide insensitive ubiquinol oxidase (Cio), are also negatively regulated by ArgR. Interestingly, expression of *argR* is enhanced up to 14-fold in cultures grown under static conditions compared to fully aerated cultures (Fig. S2). Additional cytochrome c-type proteins are among the differentially regulated functions identified. Among these, a homolog of the protein encoded by PP_3332 has been described in *P. aeruginosa* to specifically support morphology and respiration of bacterial cells in biofilms [41].

Other components involved in biofilm formation and stability are also part of the regulon: the mRNAs corresponding to the adhesin LapF (PP_0806) and the operon encoding the species-specific exopolysaccharide Pea are overrepresented in the Δ*argR* mutant, whereas the opposite is true for the cellulose biosynthesis operon. These data are in agreement with previous results obtained with transcriptional fusions [20]. It is also worth mentioning that expression of the *lapF* and *pea* promoters has been shown to be reduced in mutants affected in arginine biosynthesis [19].

**Fig. 5.**
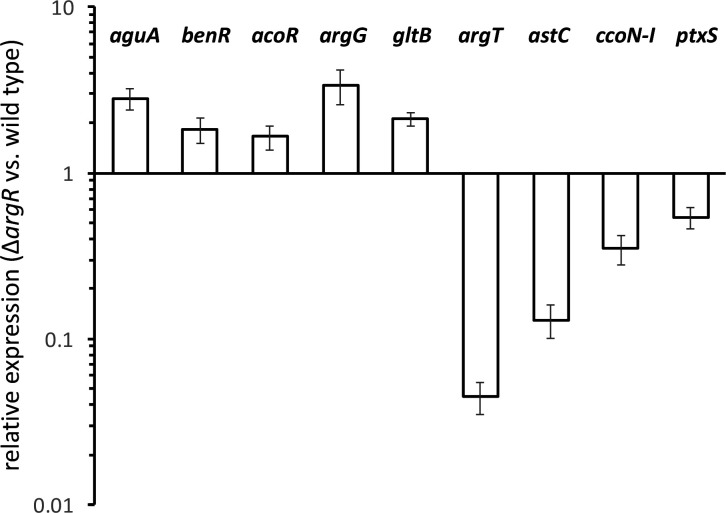
Quantitative RT-PCR of selected genes. The relative expression of the indicated genes in the Δ*argR* mutant with respect to the wild-type was analysed by qRT-PCR, as described in the Methods section. Fold-change data are presented in logarithmic scale for ease of representation, and correspond to the averages and standard deviations of three biological replicas with three technical repetitions.

Several of the identified genes were selected for quantitative RT-PCR analysis, as indicated in the Methods section. As shown in Fig. 5, the results confirm their differential expression in the Δ*argR* background with respect to the wild-type observed in the RNA-Seq analysis, showing either negative (*aguA*, *benR*, *acoR*, *argG* and *gltB*) or positive (*argT*, *astC*, *ccoN-I* and *ptxS*) regulation by ArgR. The most evident difference was observed for *argT*, a result consistent with previous evidences based on transcriptional fusions [20].

### Identification of predicted ArgR binding sites

**Fig. 6.**
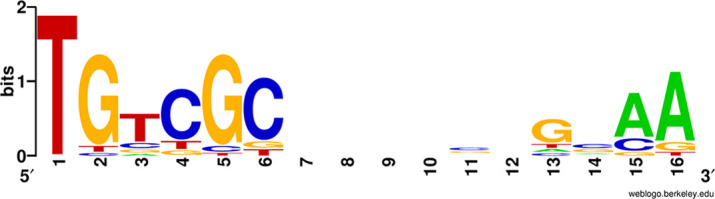
Consensus ARG box in *P. alloputida* KT2440, created with WebLogo [37] using the sequences indicated in Tables S3 and S4.

A characteristic sequence signature, named ARG box, has been defined as the recognition site for ArgR in *P. aeruginosa*. It presents the consensus 5′-TGTCGCN_6_GNAA-3′, which appears repeated in tandem with a separation of 5 bp, the second repeat generally being less conserved [21,39]. The presence of putative ARG boxes has been previously reported in KT2440 in the upstream regions of *argG*, *argT*, *astC*, and other genes [20], although the distance between the ‘half-sites’ (i.e. the ARG box consensus sequence indicated above) is rather variable. The possible existence of ARG boxes in the upstream regions of the differentially expressed transcriptional units, which could be indicative of direct binding by ArgR, was analysed. Using Virtual Footprint with the * P. aeruginosa* ArgR position-weighted matrix at the PRODORIC database [36], a total of 87 and 48 putative sites were identified in the intergenic regions upstream of genes under negative or positive regulation by ArgR, respectively (data not shown). However, the current version of this tool does not allow to define stringency parameters other than the maximum distance to the ATG or the removal of palindromic sequences, and some of the identified sites seem to diverge notably from the ARG box consensus. When sequences showing three or more mismatches with respect to the conserved bases are removed, the number of putative sites is reduced to 61 and 41, respectively (Tables S4 and S5). With the list of predicted ARG boxes, a 16 bp consensus was created (Fig. 6). The data indicate that five of the first six bases and the two A’s in positions 15 and 16 are highly conserved. In some genes there is more than one potential ARG box, but the separation between them is variable, and the 5 bp spacer proposed in *P. aeruginosa* between ARG boxes is only clearly conserved in the case of *argG*.

## Discussion

This work provides a broad overview of the impact of ArgR on the physiology of *Pseudomonas alloputida* during growth in the presence of arginine. This amino acid is not only a carbon, nitrogen and energy source, but also the precursor of other relevant metabolites in many organisms, as well as a modulator of the lifestyle of different bacteria. Its relevance in *Pseudomonas* has been recently reviewed [25], and therefore we will not delve into it here. The most noticeable result of the present work is the fact that, contrary to what has been reported in species such as *P. aeruginosa* or *E. coli*, in *P. alloputida* ArgR exerts a direct or indirect influence on expression of a wide range of genes and functions, beyond arginine metabolism, although not to the huge extent reported in *S. coelicolor*. It should be noted, however, that the genome of this microorganism is much larger than that of KT2440 (8.7 versus 6.2 Mbp, according to GenBank data). Also, no homolog of the *P. aeruginosa* DNR regulator, controlling denitrification-related functions, is present in the non-denitrifying strain KT2440, indicating fundamental differences in some aspects of nitrogen metabolism between the two species.

The AST pathway, positively controlled by ArgR, was found to be used by *P. putida* when arginine mainly serves as nitrogen supply, since the requirement of succinate to yield glutamate is thought to be wasteful under carbon-limiting conditions [42]. The ArgR metabolic re-programming occurring in the presence of both glucose and arginine also leads to the induction of the ketogluconate pathway, which contributes to sustaining the membrane potential, the intracellular reducing power and the trafficking of charged compounds [43]. This newly identified role of ArgR adds an additional level of complexity to the intricate regulatory network controlling glucose metabolism [44].

Some other aspects seem of particular interest and will deserve a more in-depth study. One is the influence of ArgR on expression of stress-related genes, with four members of the universal stress protein family being positively regulated by this transcription factor, out of the 11 annotated in the genome of KT2440. Proteins of this family have been correlated with the response of *Pseudomonas* to aromatic compounds or anaerobic conditions [45,46], but their precise role remains to be determined. A second point of interest is the differential regulation of biofilm-associated functions by ArgR. Expression of the large protein LapF, involved in cell-cell interactions [47], and the species-specific exopolysaccharide Pea are negatively regulated by ArgR, whereas the *bcs* operon, responsible for cellulose synthesis, is under positive control by this transcriptional regulator. This could be indicative of the existence of different biofilm formation strategies depending on particular environmental conditions, in this case linked to the response to arginine.

The results indicating the presence of putative ARG boxes in the upstream regions of genes identified in the RNASeq analysis will require additional experimentation to confirm direct binding of ArgR to the identified sequences. However, they leave open an intriguing question, i.e. the mechanism by which ArgR functions as a positive or negative regulator of different genes in the presence of arginine. Except for slight changes in the frequency of appearance of a certain base in some positions, no clear divergence can be observed between ARG boxes of genes up- or down-regulated in the *ΔargR* mutant (not shown). Also, the average distance to the start codon does not seem to be very different (158±90 bp vs. 148±94 bp, respectively). Identification of the transcriptional start sites and promoter regions may provide further evidence, and in any case the participation of ancillary elements cannot be discarded.

In the course of this work, we noticed the existence of some overlap between the ArgR regulon and the RoxSR regulon. This highly conserved global regulatory two-component system, also known as RegB/RegA [48], is involved in redox signalling and cytochrome oxidase activity, as well as in population density dependent regulation mediated by fatty acids in KT2440 [49,50]. A number of genes previously reported to be differentially expressed in a mutant in which the *roxSR* operon was disrupted [49], including *argT* and *argG*, showed also differential expression in the Δ*argR* mutant with respect to the wild-type strain (Table 1). These results support a connection between redox and arginine signalling, similar to what has been suggested in *P. aeruginosa* regarding the phosphodiesterase RmcA [10,51] and add weight to data on the relevance of ArgR for maintaining the metabolic activity of *P. putida* biofilms [25].

**Table 1.** Overlap between ArgR and RoxR regulons

Locus	Gene(s)	Function	ArgR	RoxR
PP_0203		dipeptidase	+	+
PP_0362	*bioB*	biotin synthesis	+	+
PP_0879	*dppB*	dipeptide transport	+	+
PP_0881	*dppD*	dipeptide transport	+	+
PP_3332		cytochrome c type protein	+	+
PP_3780		hypothetical protein	+	+
PP_3822_3823		cytochrome c type proteins	+	+
PP_4250_4251_4252_4253	*ccoNOQP-I*	cytochrome oxidase (cbb3-I)	+	+
PP_4477	*astB*	arginine catabolism	+	+
PP_4480	*astA-II*	arginine catabolism	+	+
PP_4486	*argT*	arginine transport	+	+
PP_0294	*cbcV*	choline/betaine/carnitine transport	−	+
PP_0308		dipeptidase	−	+
PP_0310	*dgcA*	dimethylglycine dehydrogenase	−	+
PP_0311_0312_0313	*dgcB*	dimethylglycine dehydrogenase; flavoprotein subunits	−	+
PP_0315	*gbcA*	glycine-betaine dioxygenase subunit	−	+
PP_0326	*soxG*	sarcosine oxidase subunit gamma	−	+
PP_0328	*fdhA*	formaldehyde dehydrogenase	−	+
PP_1088	*argG*	arginine synthesis	−	+
PP_1741	*betX*	choline/betaine/carnitine transport	−	+
PP_2184		formate dehydrogenase subunit beta	−	+
PP_0103		cytochrome c oxidase subunit (aa3)	−	−
PP_0711	*ycaC-I*	putative hydrolase	−	−
PP_0806	*lapF*	adhesin	−	−
PP_2359		putative Type one pili subunit	−	−
PP_2362		usher protein	−	−
PP_2572		hypothetical protein	−	−
PP_2941		hypothetical protein	−	−
PP_5602	*peaA*	quinohemoprotein amine dehydrogenase subunit	−	−
PP_3533		rnithine cyclodeaminase	−	−
PP_3611		hypothetical protein	−	−
PP_4055	*glgX*	glycogen debranching enzyme	−	−
PP_4851	*psiF*	phosphate starvation-inducible protein	−	−

Positive (+) or negative (-) regulation by ArgR (this work) and RoxR [49] is indicated.

The arginine deiminase (ADI) degradation pathway functions as a source of ATP from ADP and carbamoyl-phosphate independently of O_2_ [52]. In our study this pathway was found to be induced by ArgR, and expression of *argR* increased under low aeration. Remarkably, the anaerobic or low-oxygen-responsive global regulator ANR (annotated as FnrA in KT2440, corresponding to locus PP_4265) is known to directly regulate the expression of the terminal oxidases Cbb3-1 (positively) and Cio (negatively), which have high and low affinity for O_2_, respectively [53]. Moreover, aerobic electron transport chain has been reported to be part of the core of the ANR regulon in *Pseudomonas* species [54]. Likewise, we have found in this work that Cbb3-1 and Cio show positive and negative regulation by ArgR, respectively. Although further evidence is required to determine if this is a direct or indirect effect, we could speculate that ArgR and ANR interact at the promoters of these terminal oxidases, and perhaps of other bacterial determinants influenced by both regulators, such as catalase A and azurin (Tables S1 and S2; [54]), similarly to what was proposed for the *arcD* promoter in *P. aeruginosa* [55].

All these connections and their associated signalling mechanisms will deserve further exploration, but the results obtained so far already provide a glimpse of the regulatory complexity underlying the metabolic versatility of *P. alloputida* KT2440.

## supplementary material

10.1099/mic.0.001449 Uncited Supplementary Material 1.
