# Supplementary material for: Gene expression reprogramming of Pseudomonas alloputida in response to arginine through the transcriptional regulator ArgR
Source: Microbiology (Reading). 2024 Mar 21;170(3):001449. doi: 10.1099/mic.0.001449 (PMC10963909; doi:10.1099/mic.0.001449)
Supplement: Uncited Supplementary Material 1. [file mic-170-01449-s001.pdf]

SUPPLEMENTARY MATERIAL

Gene expression reprogramming of *Pseudomonas alloputida* in response to arginine through the transcriptional regulator ArgR

María Antonia Molina-Henares, María Isabel Ramos-González, Serena Rinaldo, Manuel Espinosa-Urgel

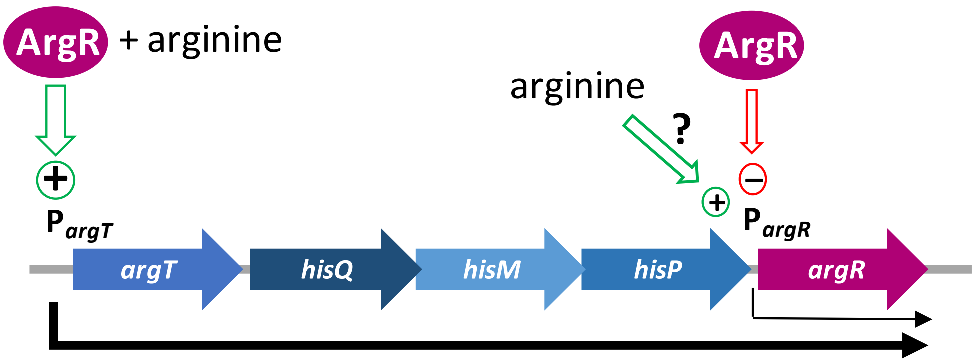

Figure S1. Overview of the *argT-hisQMP-argR* operon in KT2440 and its transcriptional regulation. The promoter upstream *argT* (P_{argT}) is fully dependent on ArgR and responds to arginine, being responsible for expression of the whole operon. A second, weaker promoter (P_{argR}) ensures basal expression of *argR* and shows a complex regulation, with ArgR exerting a negative effect on transcription and arginine stimulating it, by relieving ArgR repression and/or through other mechanism. Details can be found in reference [20] (see main text).

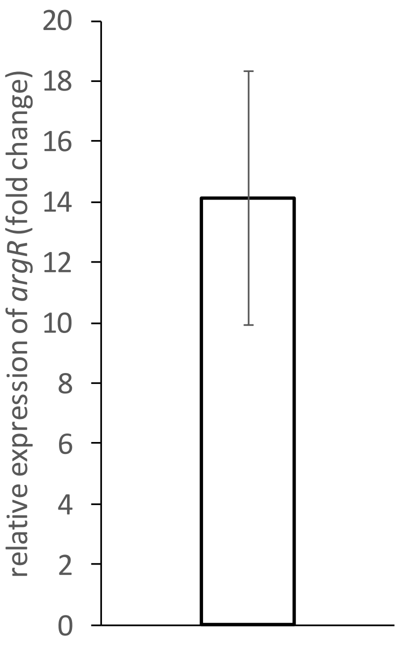

Figure S2. Expression of *argR* is increased in KT2440 cultures grown under microaerobic conditions compared with aerated cultures. Relative expression was analysed by qRT-PCR, as indicated in the Methods section. Data correspond to the calculated fold change average and the standard deviation from three biological replicas with three technical repetitions.

Table S1. Oligonucleotides used for qRT-PCR

**locus/gene**	**sequence 5´-3´**	**Fragment size (bp)**
PP_0557 *acoR*	CATGTGGACTGCGTCAGCAG<br>CTGGCGATGCGCAGGAACA	203
PP_0266 *aguA*	ATGGTTTCCACATGCCCGCC<br>TTCGGCTGGTCGAGTTGGC	208
PP_3159 *benR*	GTTTCACCACGCCGACCCTTA<br>GACTTGCAGGTGGTAGATGGTT	205
PP_3380 *ptxS*	ACGGGTCACCATCAGTGAAGTG<br>GCCACCAGCATGCCGATCA	204
PP_4481 *astC*	GCAACGTGCCGATTTCGACC<br>GACGTGCCAGAGGGTGTTG	208
PP_1088 *argG*	GCGGCCTTGATACTTCGGTGA<br>CCTTCGTAGACGGTGTTGGC	220
PP_4482 *argR*	CCAGCGAATCGGTTTTCTCATC<br>CCTGCAGGAAGACCAACTCG	125
PP_4486 *argT*	CTGGCGCTGTCTGTGTTTTCC<br>GGATCAGGCCGTCGAACTC	202
PP_4250 *ccoN-I*	ATAACTACAAGGTGGTCCGCC<br>ATAGGAGGTGGCGAACAGTGC	209
PP_5076 *gltB*	GCCTGATCGCCCATATGACG<br>CAGCTCGACAGCGAAGTGC	191

Table S2. Transcripts overrepresented (*P*-value <0.01; log_2FC>0.8; FDR<0.05) in the Δ*argR* mutant vs. wild type KT2440 (=negative regulation by ArgR). Loci predicted to form an operon are boxed, with those experimentally shown as a single transcriptional unit highlighted in yellow ([38]; see main text).

log_2FC	locus	gene	description
1.13	PP_0023		hypothetical protein
1.44	PP_0056	*betA-I*	choline dehydrogenase
2.27	PP_0057		major facilitator family transporter
1.17	PP_0086		hypothetical protein - putative flavin binding protein
0.87	PP_0101		sulfate transporter
0.90	PP_0103		cytochrome c oxidase subunit 2
0.97	PP_0104	*ctaD*	cytochrome c oxidase subunit 1
1.02	PP_0105		cytochrome c oxidase assembly protein
0.97	PP_0106		cytochrome c oxidase subunit 3
0.90	PP_0107		hypothetical protein - cytochrome c oxidase biogenesis
1.15	PP_0108		hypothetical protein
0.89	PP_0109		membrane protein - cytochrome oxidase assembly protein
1.58	PP_0155	*pntB*	pyridine nucleotide transhydrogenase subunit beta
1.66	PP_5747	*pntAB*	pyridine nucleotide transhydrogenase subunit alpha
1.62	PP_0156	*pntAA*	pyridine nucleotide transhydrogenase subunit alpha
0.91	PP_0266	*aguA*	agmatine deiminase
1.37	PP_0294	*cbcV*	choline/betaine/carnitine ABC transporter
1.19	PP_0295	*cbcW*	choline/betaine/carnitine ABC transporter
1.28	PP_0296	*cbcX*	choline/betaine/carnitine ABC transporter
1.07	PP_0308		dipeptidase
1.17	PP_0309		hypothetical protein
1.17	PP_0310	*dgcA*	dimethylglycine dehydrogenase subunit
0.98	PP_0311	*dgcB*	dimethylglycine dehydrogenase subunit
1.20	PP_0312		electron transfer flavoprotein subunit alpha
1.38	PP_0313		electron transfer flavoprotein subunit beta
0.96	PP_0315	*gbcA*	glycine-betaine dioxygenase subunit
0.85	PP_0326	*soxG*	sarcosine oxidase subunit gamma
1.59	PP_0328	*fdhA*	formaldehyde dehydrogenase
1.01	PP_0395	*ycgB*	type IV piliation protein
0.93	PP_0396		hypothetical protein
0.83	PP_0397	*yeaG*	protein kinase
1.77	PP_0544		ethanolamine transporter
2.38	PP_0552	*bdhA*	2,3-butanediol dehydrogenase
3.32	PP_0553	*acoC*	acetoin catabolism
3.45	PP_0554	*acoB*	acetoin catabolism
3.26	PP_0555	*acoA*	acetoin catabolism
2.40	PP_0556		acetoin catabolism
0.92	PP_0557	*acoR*	acetoin catabolism regulatory protein
0.83	PP_0597	*mmsA-I*	methylmalonate-semialdehyde dehydrogenase
0.97	PP_0613		amidase family protein
0.99	PP_0614		bifunctional N-carbamoyl-beta-alanine amidohydrolase/allantoine amidohydrolase
1.06	PP_0615		branched-chain amino acid transport
0.91	PP_0616		branched-chain amino acid transport
0.81	PP_0617		branched-chain amino acid ABC transporter permease
5.15	PP_0675	*gdhA*	NADP-dependent glutamate dehydrogenase
2.46	PP_0676		transcription elongation factor
0.85	PP_0711	*ycaC-I*	putative hydrolase
0.90	PP_0765		hypothetical protein
0.88	PP_0766		hypothetical protein
0.87	PP_0806	*lapF*	adhesin LapF
1.05	PP_1078		ABC transporter ATP-binding protein
1.24	PP_1079	*argF*	arginine synthesis
1.69	PP_1088	*argG*	arginine synthesis
1.08	PP_1121		OmpA family protein
1.11	PP_1122		OmpA family protein
0.90	PP_1168	*dctQ*	TRAP dicarboxylate transporter subunit DctQ
0.82	PP_1169	*dctP*	TRAP dicarboxylate transporter subunit DctP

0.88	PP_1188	*dctA-I*	C4-dicarboxylate transport protein
0.83	PP_1376	*pcaK*	4-hydroxybenzoate transporter
0.82	PP_1377	*pcaF-I*	beta-ketoadipyl-CoA thiolase
0.88	PP_1379	*pcaB*	3-carboxy-cis,cis-muconate cycloisomerase
1.11	PP_1380	*pcaD*	3-oxoadipate enol-lactonase
1.30	PP_1381	*pcaC*	4-carboxymuconolactone decarboxylase
0.86	PP_1382	*pcaP*	porin
1.01	PP_1383	*galP-II*	porin-like protein
1.04	PP_1389		oxaloacetate decarboxylase
1.00	PP_1481	*patD*	aminobutyraldehyde dehydrogenase
0.86	PP_1482	*ydcV*	polyamine ABC transporter permease
0.81	PP_1483	*ydcU*	polyamine ABC transporter permease
0.86	PP_1486	*ydcS*	polyamine ABC transporter, periplasmic polyamine-binding protein
0.98	PP_1659		hypothetical protein
1.07	PP_1660		hypothetical protein
1.05	PP_1661		dehydrogenase - GMC (glucose-methanol-choline) family oxidoreductase
1.07	PP_1662		hypothetical protein
1.01	PP_1726		ABC transporter substrate-binding protein (aminoethylphosphonate?)
1.36	PP_1741	*betX*	choline/betaine/carnitine ABC transporter substrate-binding protein
1.21	PP_1742	*yjcH*	inner membrane protein
1.45	PP_1743	*actP-I*	acetate permease
0.93	PP_1762		hypothetical protein - heme oxygenase-like superfamily
1.04	PP_2183		formate dehydrogenase subunit gamma
1.17	PP_2184		formate dehydrogenase subunit beta
1.54	PP_2185		formate dehydrogenase subunit alpha
0.89	PP_2251		membrane protein - PACE efflux transporter domain
1.07	PP_2290		hypothetical protein
0.98	PP_2291		hypothetical protein
1.22	PP_2351	*prpE*	propionyl-CoA synthetase
0.96	PP_2359		putative Type 1 pili subunit CsuA/B protein
0.87	PP_2360		type I pili subunit CsuA/B
0.98	PP_2361		chaperone protein
1.02	PP_2362		usher protein
0.84	PP_2512	*folEA-II*	GTP cyclohydrolase I
0.82	PP_2563		antibiotic biosynthesis protein
1.01	PP_2572		hypothetical protein
0.94	PP_2573		hypothetical protein
1.03	PP_2576	*(pqiA)*	pseudogene?
0.97	PP_2577	*pqiA*	paraquat-inducible protein
0.93	PP_2578		hypothetical protein-paraquat inducible
1.22	PP_2602	*ligC*	4-carboxy-2-hydroxymuconate-6-semialdehyde dehydrogenase
1.51	PP_2603		hypothetical protein
1.10	PP_2643	*pcaY*	aromatic acid chemoreceptor
2.03	PP_2663		hypothetical protein, FIST domain
1.58	PP_2664		two-component system sensor histidine kinase/response regulator
1.28	PP_2665	*agmR*	glycerol metabolism activator
1.47	PP_2666		hypothetical protein - PQQ-dependent catabolism-associated CXXCW motif protein
1.17	PP_2667	*pedC*	transporter (alcohol?)
2.08	PP_2668	*pedB*	transporter (alcohol?)
2.25	PP_2669	*pedA2*	
2.00	PP_5538	*pedA1*	transporter, substrate-binding protein
1.48	PP_2671	*pedS2*	sensor histidine kinase
1.36	PP_2672	*pedR2*	Transcriptional regulator
0.97	PP_2674	*qedHA*	renamed pedE. Quinoprotein ethanol dehydrogenase
2.64	PP_2675		cytochrome c-type protein
2.20	PP_2676		substrate-binding protein
1.96	PP_2677		hypothetical protein - quinoprotein dehydrogenase-associated SoxYZ-like carrier
1.27	PP_2678		hydrolase
2.40	PP_2679	*qedHB*	renamed pedH. Quinoprotein ethanol dehydrogenase
1.40	PP_2680	*aldB-II*	aldehyde dehydrogenase
2.12	PP_2681	*pqqD-II*	coenzyme PQQ synthesis protein D
0.84	PP_2706		hypothetical protein

0.83	PP_2733		membrane protein
0.94	PP_2734	*cfa*	cyclopropane-fatty-acyl-phospholipid synthase
0.93	PP_2796		hypothetical protein
0.85	PP_2797	*actP-II*	acetate permease
0.81	PP_2911	*gabP-III*	gamma-aminobutyrate permease
0.91	PP_2925	*mqo-III*	malate:quinone oxidoreductase
0.83	PP_2941		hypothetical protein
0.92	PP_2945		two-component system sensor histidine kinase/response regulator
0.96	PP_2964		transposase
0.85	PP_2966		hypothetical protein
0.89	PP_3135		glycosyl transferase
0.85	PP_3136		O-acetyltransferase. Pea synthesis cluster
0.84	PP_3137		group 2 family glycosyl transferase. Pea synthesis cluster
0.81	PP_3138		VirK domain protein (part of the Pea gene cluster)
1.01	PP_3145		hypothetical protein
1.10	PP_3146		oxidoreductase
1.29	PP_3147	*potF-II*	putrescine-binding protein
1.33	PP_3148		glutamine synthetase
0.99	PP_3159	*benR*	BenABC operon transcriptional activator
1.15	PP_3161	*benA*	benzoate 1,2-dioxygenase subunit alpha
1.10	PP_3162	*benB*	benzoate 1,2-dioxygenase subunit beta
1.35	PP_3163	*benC*	benzoate 1,2-dioxygenase electron transfer component
1.67	PP_3164	*benD*	1,6-dihydroxycyclohexa-2,4-diene-1-carboxylate dehydrogenase
2.35	PP_3165	*benK*	benzoate MFS transporter
2.14	PP_3166	*catA-II*	catechol 1,2-dioxygenase
2.55	PP_3167	*benE-II*	benzoate transport protein
2.87	PP_3168	*nicP-I*	porin-like protein
1.32	PP_3169		membrane protein
1.85	PP_3352	*atsA*	arylsulfatase
1.39	PP_3353		sulfatase-modifying protein
0.89	PP_3449		hypothetical protein
0.83	PP_3460	*peaC*	quinohemoprotein amine dehydrogenase subunit gamma
0.84	PP_5602	*peaA*	quinohemoprotein amine dehydrogenase subunit alpha
0.95	PP_3504		hypothetical protein
1.27	PP_3533		ornithine cyclodeaminase
1.06	PP_3611		hypothetical protein
0.95	PP_3668	*katG*	catalase-peroxidase
0.89	PP_3723	*aruI*	2-ketoarginine decarboxylase
2.15	PP_3724		acyl-CoA synthetase
2.24	PP_3725		acyl-CoA dehydrogenase
1.88	PP_3726		enoyl-CoA hydratase/isomerase family protein
2.70	PP_3727	*rocE*	amino acid permease RocE
0.82	PP_3746	*glcE*	glycolate oxidase FAD-binding subunit
1.00	PP_3954		substrate-binding protein
1.17	PP_3955		TRAP transporter permease
0.97	PP_4011	*icd*	NADP(+)-specific isocitrate dehydrogenase
1.04	PP_4021	*cpo*	non-heme chloroperoxidase
0.83	PP_4033	*rnz*	ribonuclease Z
1.06	PP_4034	*hyuC*	bifunctional N-carbamoyl-beta-alanine amidohydrolase/allantoine amidohydrolase
0.97	PP_4035	*pydP*	NCS1 family transporter PydP
0.84	PP_4036	*pydB*	bifunctional D-hydantoinase/dihydropyrimidinase
1.22	PP_4037	*pydX*	NADP-dependent dihydropyrimidine dehydrogenase subunit
1.18	PP_4038	*pydA*	NADP-dependent dihydropyrimidine dehydrogenase subunit PreA
0.81	PP_4055	*glgX*	glycogen debranching enzyme
0.82	PP_4058	*glgB*	1,4-alpha-glucan branching enzyme
0.81	PP_4111	*fusB*	elongation factor G 2
0.81	PP_4137		outer membrane siderophore receptor
0.85	PP_4138	*chrR*	chromate reductase
0.93	PP_4255	*ccoN-II*	cbb3-type cytochrome c oxidase subunit 1
1.09	PP_4256	*ccoO-II*	cbb3-type cytochrome c oxidase subunit 2
1.02	PP_4257	*ccoQ-II*	cbb3-type cytochrome c oxidase subunit 3
0.98	PP_4491	*phhB*	pterin-4-alpha-carbinolamine dehydratase

0.93	PP_4524		sodium-solute symporter
1.36	PP_4578		MFS transporter
0.90	PP_4650	*cioB*	cyanide insensitive ubiquinol oxidase subunit II
1.10	PP_4659	*ggt*	gamma-glutamyltranspeptidase
0.83	PP_4748		amino acid ABC transporter substrate-binding protein
0.82	PP_4750		amino acid ABC transporter permease
0.98	PP_4751		amino acid ABC transporter ATP-binding protein
0.87	PP_4752		Xaa-Pro aminopeptidase
0.83	PP_4851	*psiF*	phosphate starvation-inducible protein PsiF
1.11	PP_4863	*livF-II*	ABC transporter ATP-binding protein
0.95	PP_4864	*braF*	ABC transporter ATP-binding protein
0.97	PP_4865	*braE*	high-affinity branched-chain amino acid ABC transporter permease
1.07	PP_4867	*ilvJ*	high-affinity branched-chain amino acid ABC transporter permease
1.65	PP_5075	*gltD*	glutamate synthase subunit beta
1.81	PP_5076	*gltB*	glutamate synthase subunit alpha
0.93	PP_5173		RND family transporter
0.97	PP_5313	*hupA*	DNA-binding protein HU-alpha
2.29	PP_5338	*aspA*	aspartate ammonia-lyase
1.61	PP_5340	*aphA*	acetylpolyamine aminohydrolase
1.34	PP_5341	*potF-IV*	putrescine-binding protein

FC: fold change; FDR: false discovery rate

Table S3. Transcripts underrepresented (*P*-value <0.01; log_2FC<-0.8; FDR<0.05) in the Δ*argR* mutant vs. wild type KT2440 (=positive regulation by ArgR). Loci predicted to form an operon are boxed, with those experimentally shown as a single transcriptional unit highlighted in yellow ([38]; see main text).

log_2FC	locus	gene	description
-0.84	PP_0125		cytochrome c-type protein
-1.61	PP_0203		dipeptidase
-0.85	PP_0240	*ssuB*	aliphatic sulfonates ABC transporter ATP-binding protein
-1.28	PP_0273		hypothetical protein
-2.02	PP_0280		arg transport
-2.24	PP_0281		arg transport
-1.48	PP_0282	*artJ*	arg transport
-1.77	PP_0283	*aotP*	arg transport
-1.45	PP_0362	*bioB*	biotin synthesis
-1.58	PP_0363	*bioF*	biotin synthesis
-0.96	PP_0364	*bioH*	biotin synthesis
-0.87	PP_0365	*bioC*	biotin synthesis
-0.93	PP_0481	*katA*	catalase
-3.02	PP_0878	*dppF*	dipeptide transport
-3.08	PP_0879	*dppD*	dipeptide transport
-3.36	PP_0880	*dppC*	dipeptide transport
-3.74	PP_0881	*dppB*	dipeptide transport
-4.60	PP_0882	*dppA-I*	dipeptide transport
-5.46	PP_0883	*opdP*	dipeptide transport
-4.21	PP_0884	*dppA-II*	dipeptide transport
-2.42	PP_0885	*dppA-III*	dipeptide transport
-1.11	PP_0987	*tdcG-II*	L-serine dehydratase
-1.22	PP_0988	*gcvP-I*	glycine dehydrogenase
-0.84	PP_0989	*gcvH-I*	glycine cleavage system protein H
-0.94	PP_0998		hypothetical protein
-1.03	PP_0999	*arcC*	arg catabolism
-1.18	PP_1000	*arcB*	arg catabolism
-0.99	PP_1001	*arcA*	arg catabolism
-1.09	PP_1130		hypothetical protein
-1.12	PP_1153		lipoprotein
-0.89	PP_1249		DUF4223 domain-containing protein
-1.03	PP_1297	*yhdW*	amino acid transport
-1.28	PP_1298	*yhdX*	amino acid transport
-1.12	PP_1299	*yhdY*	amino acid transport
-1.03	PP_1300	*yhdZ*	amino acid transport
-0.97	PP_1395		AraC family transcriptional regulator
-2.10	PP_1396		hypothetical protein
-1.55	PP_1397		hypothetical protein
-0.81	PP_1399		hypothetical protein
-1.67	PP_1400	*kgtP*	alpha-ketoglutarate permease
-0.82	PP_1535		putative methyltransferase
-0.91	PP_5472		hypothetical protein
-0.96	PP_5473		LuxR family transcriptional regulator
-0.87	PP_1691		hypothetical protein
-3.41	PP_2080	*gdhB*	NAD-specific glutamate dehydrogenase
-0.81	PP_2121		lipoprotein
-1.07	PP_2256		Cro/CI family transcriptional regulator
-0.84	PP_2629		hypothetical protein
-1.65	PP_2631		cellulose biosynthesis protein BcsF/YhjT
-0.94	PP_2632	*bcsG*	cellulose biosynthesis
-0.97	PP_2634	*bcsQ*	cellulose biosynthesis
-1.20	PP_2637	*bcsZ*	cellulose biosynthesis
-1.16	PP_2638		cellulose synthase operon protein C
-0.86	PP_2646		hypothetical protein
-1.35	PP_2648		universal stress protein family protein
-0.82	PP_2652		hydratase/decarboxylase
-0.97	PP_2745		universal stress protein family protein

-1.04	PP_2753		ABC transporter ATP-binding protein
-1.19	PP_2874		hypothetical protein
-0.87	PP_3180		Smp-30/Cgr1 family protein (putative gluconolactonase)
-0.95	PP_3190		ornithine cyclodeaminase/mu-crystallin family protein
-1.18	PP_3191		threonine ammonia-lyase/dehydratase
-1.16	PP_3231		hypothetical protein
-1.90	PP_3232		acetyltransferase
-1.69	PP_3234		HSP20 family heat shock protein
-1.41	PP_3235		hypothetical protein
-1.25	PP_3236		lipoprotein OprI
-1.14	PP_3237		universal stress protein family protein
-1.12	PP_3238		transcriptional regulator PyrR
-1.03	PP_3289		acetyltransferase
-0.90	PP_3290		universal stress protein family protein
-0.99	PP_3291		metallo-beta-lactamase family protein
-2.59	PP_3332		cytochrome c-type protein
-2.41	PP_3333		hypothetical protein
-1.95	PP_5743		TonB-dependent receptor protein
-1.34	PP_3334		hypothetical protein
-0.85	PP_3336		hypothetical protein
-2.87	PP_3374		hypothetical protein
-1.60	PP_3375	*endA*	endonuclease I
-1.28	PP_3376	*ptxD*	phosphonate dehydrogenase
-1.73	PP_3377	*kguT*	2-ketogluconate transporter, putative
-2.61	PP_3378	*kguK*	2-ketogluconokinase
-2.25	PP_3379	*kguE*	epimerase
-1.10	PP_3380	*ptxS*	2-ketogluconate utilization repressor
-1.39	PP_3382	*gadA*	gluconate 2-dehydrogenase cytochrome c subunit
-1.51	PP_3383	*gadB*	gluconate 2-dehydrogenase flavoprotein subunit
-1.50	PP_3384	*gadC*	gluconate 2-dehydrogenase gamma subunit
-1.63	PP_3621	*iorAB*	isoquinoline 1-oxidoreductase subunit alpha
-1.66	PP_3622	*iorBB*	isoquinoline 1-oxidoreductase subunit beta
-1.61	PP_3623	*adhB*	alcohol dehydrogenase cytochrome c subunit
-0.87	PP_3780		hypothetical protein
-1.25	PP_3822		cytochrome c family protein
-1.14	PP_3823		cytochrome c-type protein
-0.85	PP_3839	*adhP*	alcohol dehydrogenase
-0.83	PP_3929		hypothetical protein
-1.14	PP_4184	*brnQ*	branched-chain amino acid transporter
-1.30	PP_4250	*ccoN-I*	cbb3-type cytochrome c oxidase subunit
-1.52	PP_4251	*ccoO-I*	cbb3-type cytochrome c oxidase subunit
-1.67	PP_4252	*ccoQ-I*	cbb3-type cytochrome c oxidase subunit
-1.72	PP_4253	*ccoP-I*	cbb3-type cytochrome c oxidase subunit
-2.02	PP_4475	*astE*	arg catabolism
-2.20	PP_4476		hypothetical protein
-3.27	PP_4477	*astB*	arg catabolism
-3.86	PP_4478	*astD*	arg catabolism
-3.94	PP_4479	*astA-I*	arg catabolism
-3.79	PP_4480	*astA-II*	arg catabolism
-2.79	PP_4481	*astC*	arg catabolism
-9.85	PP_4482	***argR***	
-4.00	PP_4483	*hisP*	arg transport
-3.93	PP_4484	*hisM*	arg transport
-4.11	PP_4485	*hisQ*	arg transport
-4.17	PP_4486	*argT*	arg transport
-0.92	PP_4870		azurin
-1.06	PP_5343		putative transcriptional regulator
-1.07	PP_5344		acetyltransferase
-2.60	PP_5461		hypothetical protein
-0.90	PP_mr39		ncRNA mr39
-1.09	PP_mr42		ncRNA mr42
-0.90	PP_t34		tRNA Asp (GTC)

Table S4. Putative ARG boxes in genes negatively regulated by ArgR. Genes related to arginine transport or metabolism are shown in boldface.

Locus (Gene)	Distance to ATG	Predicted binding sequences	
PP_0056 (*betA-I*)	-64,-298	TGTTTCATCCACGAAA	TGGCGGCGGTCAGCAA
PP_0295 (*cbcW*)	-256	TGTCGTTCGAGCTCAA	
PP_0296 (*cbcX*)	-275	TGTTCTCAACCAGAAA	
PP_0308	-125	TGACGCTTTCGGCAAA	
PP_0309	-92	TGTCTGGGGCGAGTAA	
PP_0312	-49	TGTCCCAAAGCCGCAA	
PP_0313	-103,-271	TGTCGGTGATTGGCGA	TGCCGCGCAACCGCCA
PP_0328 (*fdhA*)	-130	TGTCGCTCCCAGTCAG	
PP_0555 (*acoA*)	-182	TGGCGCTGGATGGCGA	
PP_0556	-152	TGTCTCGGCAACGCAG	
PP_0557 (*acoR*)	-73	TGTCGGGGCATGCCCA	
PP_0615	-288	TGTCGCACGGCCAGCA	
PP_0711 (*ycaC-I*)	-279	TGTCGTTGCTGGCGAA	
PP_0765	-93	TGTCGCCGCAGGTGCA	
**PP_1079 (*argF*)**	-208	TGTAGCAAATAAAGAA	
**PP_1088 (*argG*)**	-76,-97	TGTCGCCCCACTGTAA	TGTCGCTTTGTCGTCA
PP_1121	-123	TGCGGCGTGCGGGCAA	
PP_1376 (*pcaK*)	-251	TGTCGTGCCGGGTGAA	
PP_1380 (*pcaD*)	-2,-294	TGTGGCGCACTTGCAA	TGTCGCTGAAATGGCG
PP_1383 (*galP-II*)	-289,-235	TGTTGCAAAAGCAGAA	TGCCGGCTGGGCGCAA
PP_1486 (*ydcS*)	-201	TGTCTCCACGTAAGAA	
PP_1661	-221	TGTGGCTGCTTGGCAG	
PP_1741 (*betX*)	-96,-48	TGTCGCATATGGGGCG	
PP_1762	-40	TTTCGCTAGTTGGAAA	
PP_2183	-248	TGATGCCGGGGCGTAA	
PP_2351 (*prpE*)	-42	TTTCGCCCTTCAATAA	
PP_2359	-95	TGTGGTAGTGGCGCAA	
PP_2362	-188,-255	TGTGGTCGTGCAGCAA	TGACGGTGCAGGGCAA
PP_2603	-157	TGTTGCCCGGGGTGAA	
PP_2666	-256	TGTCGCGATTGGGCCG	
PP_2669	-240	TCGCGCGCTGCAGCAA	
PP_2674 (*qedH-I*)	-113	TGTGGCGAAGGGCCAA	
PP_2677	-289	TTTCGCTGCCATGCAG	
PP_2680 (*aldB-II*)	-37	TGTCGTGGGACAGGCA	
PP_2941	-175	TGGTGCTTGCGCGCAA	
PP_3135	-40	TGGCGCAAAGACGTCA	
PP_3162 (*benB*)	-191	TGTCCCGTGGCGCGAA	
PP_3164 (*benD*)	-24	TCTCGCGAACTGGAGA	
PP_3168 (*nicP-I*)	-263	TGGCGCCATCAGCGAA	
PP_3352 (*atsA*)	-164	TGCCGTGTACAAGGAA	
PP_3353	-92	TGCTGCGCGACTGGAA	
PP_3460 (*peaC*)	-224	TGTCGCAGCGCCTGGA	
PP_3533	-229	TCCCGCAACAGTGCAA	
PP_4011 (*icd*)	-74	TGTCGCATGCCTTGCA	
PP_4037 (*pydX*)	-38	TGTCGCCTGCACCAAA	
PP_4255 (*ccoN-II*)	-233	TGTCGCCGCCCCAGAA	
PP_4256 (*ccoO-II*)	-215,-61	TGTGGCGCGCCATCAA	TGCCGCCAACCCGGCA
PP_4578	-22	TGTCGCCCATAAAACA	
PP_4748	-117	TGTGGCAGCGCTGGAA	
PP_4752	-218	TGTCGGCAAGCCGCAA	
**PP_5076 (*gltB*)**	-113	TGTCGCTGTGCAACAA	
PP_5173	-219	TGTCCCCGCGCAGCGA	
PP_5313 (*hupA*)	-209	TGGCGCTGAATCGTCA	
PP_5338 (*aspA*)	-37	TGGCGCCATTCCGGCA	

Table S5. Putative ARG boxes in genes positively regulated by ArgR. Genes related to arginine transport or metabolism are shown in boldface.

Locus (Gene)	Distance to ATG	Predicted binding sequences	
PP_0203	-36	TTTCCCGAACGGGTAA	
PP_0273	-154	TGTCCCGGCGCACCAA	
**PP_0283 (*aotP*)**	-45	TGGCGCACGCCAGGAA	
PP_0365 (*bioC*)	-279	TGTCGGCGTGCCGGAA	
PP_0879 (*dppD*)	-4,-281	TGCCGCCTGAGGAGAA	TGTCGCCGGCCCGTGT
PP_0884 (*dppA-II*)	-75	TGTCGTCCTGCGGCCA	
PP_0989 (*gcvH-I*)	-241	TTTCGCTACGTCGTAG	
**PP_0999 (*arcC*)**	-238	AGTCGCCAGCGTGCAT	
**PP_1001 (*arcA*)**	-69	TGGCGCCCTGGTGGCA	
PP_1249	-118	TGTTGCACAGTTGCAA	
PP_1297 (*yhdW*)	-256	TGTCGCAACGATACAA	
PP_1299 (*yhdY*)	-64	TTTCGCTGCTGATGAA	
PP_1300 (*yhdZ*)	-271	TGCCGCAGGCGCTGAA	
PP_1396	-118	TGCCGCGCTGCAGGAT	
**PP_2080 (*gdhB*)**	-187	TGTCGCAGCGCTGCAA	
PP_2121	-235	TGTCGCTGACCGTGAG	
PP_2638	-23,-83	TGTTGCCGGCCTGGAG	TGGCGCTGTTCGGCCA
PP_2646	-246,-117	TGCTGCGCACTCGCAA	TGCCGCTGCCGTGGGA
PP_2648	-250	TGTTGCAGGGCCGACA	
PP_3180	-285	TGTTGCGCAGAAGAAG	
PP_3191	-175	TGTCGCTGCAGACCCT	
PP_3289	-97	TGATGCAGATCAGAAA	
PP_3380 (*ptxS*)	-39	TTTTGCTGTATTGAAA	
PP_3621 (*iorA-II*)	-19	TGACGGGCAGGTGAAA	
PP_3623 (*adhB*)	-195	TGCCGCCTTCGCGGAT	
PP_4251 (*ccoO-I*)	-214	TGTGGCGCGCGGTCAA	
PP_4477 (*astB*)	-299	TGCCGCCATCGATGAA	
PP_4478 (*astD*)	-122,-10	TGTCGCATCACTGCCG	TGTCGGCCAGCCGGGA
PP_4481 (*astC*)	-129,-78	TGTCGCATTGCCGAAA	TGTCGCTTGGCCGCAA
PP_4483 (*hisP*)	-242,-299	TGCCGCAGTACAGCAA	TGTCGCGCATGAAAAT
PP_4484 (*hisM*)	-116	TGACGCCACCCGCGAA	
**PP_4486 (*argT*)**	-148,-117	TGTCGCTTTGAAGAAA	TGTCGTTAAAATGCCA
PP_4870	-21,-59	TCTCGCAATTGATAAA	TGACCATATCAGCAA
